# Supplementary material for: Porcine Lawsonia intracellularis Ileitis in Italy and Its Association with Porcine Circovirus Type 2 (PCV2) Infection
Source: Animals (Basel). 2023 Mar 26;13(7):1170. doi: 10.3390/ani13071170 (PMC10093578; doi:10.3390/ani13071170)
Supplement: Supplementary file 1 [file animals-13-01170-s001.zip › animals-2285193-supplementary.pdf]

Table S1. Results of histology, immunohistochemistry and microbiological examinations.

ID	Age (days)	Score			*L. intracellularis* diagnosis		PCV2 diagnosis	*Brachyspira hyodysenteriae* diagnosis	*Brachyspira pilosicoli* diagnosis
		Clinical[a]	Inflammatory infiltrate[b]	Crypt hyperplasia[c]	PCR	IHC[d]	IHC[e]	PCR	PCR
1	≤100	A	3	3	+	1	2	-	-
2	>100	A	3	2	+	3	0	-	-
3	>100	A	3	3	+	3	0	-	-
4	≤100	A	3	3	+	1	2	-	-
5	>100	C	3	3	+	1	0	-	-
6	≤100	C	3	3	+	2	1	-	-
7	≤100	C	3	3	+	1	1	-	-
8	≤100	C	3	3	+	1	0	-	-
9	>100	C	3	3	+	1	0	-	-
10	>100	C	3	3	+	1	0	-	-
11	>100	C	0	1	-	0	0	-	-
12	>100	C	2	2	-	0	0	-	-
13	>100	C	2	1	-	0	0	-	-
14	≤100	C	3	3	-	0	0	-	-
15	≤100	C	3	3	-	0	0	-	-
16	≤100	C	3	2	-	0	0	-	-
17	≤100	C	3	2	-	0	0	-	-
18	>100	C	1	2	-	0	0	-	-
19	>100	C	1	1	-	0	0	-	-
20	>100	C	3	1	+	0	0	-	-
21	>100	C	3	2	+	0	0	-	-
22	>100	C	2	2	+	2	0	-	-
23	>100	C	1	1	+	3	0	-	-
24	≤100	C	2	2	+	1	0	-	-
25	≤100	C	2	2	+	1	0	-	-
26	≤100	C	2	2	+	1	0	-	-
27	≤100	C	2	2	+	1	0	-	-
28	≤100	C	1	1	+	0	0	-	-

Note:
[a] A: acute case, C: chronic case; [b] 0: normal, 1: mild cellular infiltrate predominantly lymphohistiocyte-like, 2: moderate infiltrate with submucosa involvement, 3: severe cellular infiltrate of mucosa and submucosa; [c] 0: normal, 1: mild crypt hyperplasia, 2: moderate hyperplasia, 3: severe crypt hyperplasia with or without crypt herniation in the submucosa; [d] 0: no signal, 1: focal signal, 2: moderate multifocal signal, 3: extensive signal; [e] 0: negative; 1: cells with PCV2 antigen staining in less than 10% of lymphoid follicles; 2: cells with PCV2 antigen staining in 10-50% of lymphoid follicles; 3: cells with positive staining for PCV2 antigen in more than 50% of lymphoid follicles.
